# An novel frequent probability pattern mining algorithm based on circuit simulation method in uncertain biological networks

**DOI:** 10.1186/1752-0509-8-S3-S6

**Published:** 2014-10-22

**Authors:** Jieyue He, Chunyan Wang, Kunpu Qiu, Wei Zhong

**Affiliations:** 1 School of Computer Science and Engineering, Key Lab of Computer Network & Information Integration, MOE, Southeast University, Nanjing, 210018, China; 2 Division of Mathematics and Computer Science, University of South Carolina Upstate 800 University Way, Spartanburg, SC 29303, USA

## Abstract

**Background:**

Motif mining has always been a hot research topic in bioinformatics. Most of current research on biological networks focuses on exact motif mining. However, due to the inevitable experimental error and noisy data, biological network data represented as the probability model could better reflect the authenticity and biological significance, therefore, it is more biological meaningful to discover probability motif in uncertain biological networks. One of the key steps in probability motif mining is frequent pattern discovery which is usually based on the possible world model having a relatively high computational complexity.

**Methods:**

In this paper, we present a novel method for detecting frequent probability patterns based on circuit simulation in the uncertain biological networks. First, the partition based efficient search is applied to the non-tree like subgraph mining where the probability of occurrence in random networks is small. Then, an algorithm of probability isomorphic based on circuit simulation is proposed. The probability isomorphic combines the analysis of circuit topology structure with related physical properties of voltage in order to evaluate the probability isomorphism between probability subgraphs. The circuit simulation based probability isomorphic can avoid using traditional possible world model. Finally, based on the algorithm of probability subgraph isomorphism, two-step hierarchical clustering method is used to cluster subgraphs, and discover frequent probability patterns from the clusters.

**Results:**

The experiment results on data sets of the Protein-Protein Interaction (PPI) networks and the transcriptional regulatory networks of *E. coli *and *S. cerevisiae *show that the proposed method can efficiently discover the frequent probability subgraphs. The discovered subgraphs in our study contain all probability motifs reported in the experiments published in other related papers.

**Conclusions:**

The algorithm of probability graph isomorphism evaluation based on circuit simulation method excludes most of subgraphs which are not probability isomorphism and reduces the search space of the probability isomorphism subgraphs using the mismatch values in the node voltage set. It is an innovative way to find the frequent probability patterns, which can be efficiently applied to probability motif discovery problems in the further studies.

## Background

In the field of bioinformatics, many types of data are present as the topological graph, such as protein interaction network whose nodes represent proteins, and edges represent the interactions between proteins. Milo in 2002 proposed the concept of biological motif [1] on Science, which discussed a substructure that appears in different parts of a network, and appears significantly more frequently than in a random network. Research shows that the motif recognition is important for many biological studies. For example, motif recognition helps to study the biological network structure, function modules and evolutionary process of organisms and so on. So a lot of research on the exact network model was proposed and these researches have made some progress [1-8]. As the life process itself is a dynamic process, the motif of same function may be made up of the subgraphs which may slightly differ in topology, so Berg etc [9] proposed probability motif mining algorithms in the biological network. And Rui etc [10,11] also discussed science graph data obtained with the inevitable experimental error or noisy data, and some biological network data with probability information. Meanwhile, since biological evolution itself is a mutant selection process, the input of biological networks should also be probabilistic networks. Therefore, it is more intuitively and practically significant to mine probability motif in the probability biological network.

Most research on motif mining mainly focuses on exact graph while fewer papers work on probability motif. In the paper [11], Rui proposed to use the EM algorithm to estimate the relevant parameters for the probability motif. In this algorithm, the uncertain graph is converted to the certain graph. Since this conversion process requires a large amount of computation, this algorithm has low computational efficiency. In 2009, Rui[10] used the Bayesian model and GIBBS sampling strategy to solve the probability mult-motif. But the probability network still needs to be converted into a certain subgraph and randomly certain graph as background. As a result, the computational cost of this algorithm is still very high.

Probability Motif detection in networks consists of two main steps: 1) calculating the number of occurrences of a probability subgraph in the network and 2) evaluating the probability subgraph, which occurs significantly more frequently than in a random network. So, frequent probability pattern recognition in biological networks is an important step in identifying the probability motif. Currently, the research related to mining frequent subgraph in graph data has made a lot of process, such as gSpan [12], FFSM [13], etc...However, these researches mainly pay attention to certain graph, edges or nodes which are represented by the presence or absence. Therefore, existing frequent pattern identification algorithms for certain graph cannot be applied to frequent pattern identification of biological probability network.

On the other hand, in the uncertain data mining field, the research also has made a lot of achievements in recent years, such as uncertain data modelling and management work [14,15], and paper [16] introduced the latest technology related to uncertain data, but these studies still primarily focus on traditional uncertain data items. Research on uncertain graph has just begun, which include most reliable subgraph discovering [17-19], efficient TOP-K query [20] and other topics in the uncertain graph. Zou[21-23] also proposed some effective algorithms in mining uncertain graph frequent patterns. However, the above mentioned algorithms mainly use the possible world model. Possible world models are widely used to model uncertain data sets, in which probability graph will be converted into the corresponding possible worlds model graph that it infers, and then each probability subgraph is mapped into 2^n ^( n is the number of edges of probability subgraph) possible graph instances using the topology graph mining algorithm [12,13,24,25]. The enumerated space of probability graph instances may grow exponentially, resulting in very high algorithm complexity. So, Paper [26] firstly ignores the weight of edges in probabilistic networks and carries out the subgraph isomorphism, and then combines the random walk model to find maximal frequent subgraph, however, some of frequent probability graph in this work may be ignored.

Frequent probability pattern mining, a key step in the probability motif identification, is based on the method of probability isomorphic evaluation. Therefore, a novel method for frequent probability pattern mining in biological uncertain networks based on circuit simulation is proposed in this paper. Firstly, the partition based efficient search is applied to non-treelike subgraph mining where the probability of occurrence in random networks is small. In the second step, exact graph isomorphism identification based on circuit simulation [27] is modified to make efficient probability graph isomorphic decision. The probability graph isomorphic decision combines the analysis of circuit topology structure with related physical properties of voltage in order to directly evaluate the probability isomorphism between probability subgraphs. This innovative approach can effectively avoid the traditional method utilizing the possible world model and excludes most of subgraphs which are not probability isomorphism and reduces the search space of the probability isomorphism subgraphs by the mismatch value of node voltage set. Finally, based on the algorithm of probability subgraph isomorphism, two-step hierarchical clustering method is used to cluster subgraphs, and discover frequent probability patterns from the clusters. The experimental results on data sets of the Protein-Protein Interaction (PPI) networks and the transcriptional regulatory networks of *E. coli S.cerevisiae *show that the method can efficiently discover the frequent probability subgraphs which contain the probability motifs found in other related experiments. And, it is an innovative way to find the frequent probability patterns, which may be efficiently used for discovering probability motifs in the further studies.

The main contribution of this paper is as follows:

(1). A new algorithm of probability isomorphic decision based on circuit simulation is proposed. This approach simplifies the process of finding the probability subgraph by converting these graphs into their inferring certain graph based on the possible world model. It is an innovative way to determine the two graph probability isomorphism by comparing their nodes voltages sequence instead of the topological alignment of subgraph isomorphism, and the algorithm reduces the search space of the probability isomorphism subgraphs using the mismatch values of the node voltage set. In the narrowed set of subgraphs, the mismatch values of the subgraphs are calculated by the enumeration method.

(2). Traditional certain graph alignment is usually based on Star-alignment which needs to traverse all nodes of graph as the centre graph, resulting in high computational costs. Two-step hierarchical clustering for calculating multi-graph probability alignment is proposed to effectively reduce the computational complexity.

Briefly then, the outline of this paper is as follows. In the method section, probability isomorphic algorithm is described in details and the two-step hierarchical clustering method for discovering frequent probability pattern is introduced. In the result section, the experimental results are presented. Finally, suggestions for future work are made in the conclusion section.

## Methods

### Problem Definitions

**Definition 1 **(biological probabilistic networks): Denoted as

*g_B _*= (*V, E, Σ, L, p*),Where *V *is the node set of biological probabilistic networks *g_B_, E *⊆*V *× *V *is the set of edges of the graph *g_B_, Σ *is a set of node label, *L *:*V *→ *E *is a node labelling function, *p *: *E *→ (0,1] indicates the probability of edges in biological probabilistic networks.

Generally, the data obtained by biological experiments carry some inevitable noisy data, while biological evolution itself is a variable selection process. As a result, the concept of probability can be introduced in the definition to indicate the uncertainty of biological data.

**Definition 2 **(Probability graph isomorphism): Set the number of nodes as *k *of the two probability graph *g *= (*V, E, Σ, L, p*), *g′ *= (*V′, E′, Σ', L′, p′*), there exists a node mapping sequence <*Inje, Inje*' >, so that the two graphs have similar topology (does not require identical topology), while the absolute value of the difference between the weights of corresponding edges between nodes |Δ*_ij_*| ≤ *α*, the sum of all the absolute value of the difference between the weights of the corresponding edges $\sum_{i=1,j=1}^{k}|{\text{Δ}}_{ij}|\leq \theta$, then *g *and *g*′ is called probability isomorphic.

In other words, suppose *d *and *d*′ are the adjoin matrix of the probabilistic graph *g *and *g*′, there exist a node mapping sequence <*Inje, Inje*' >, making the following conditions satisfied:

1) $\forall i,j\in \left\{1,...,k\right\}{\text{Δ}}_{ij}=|p\left(Inje\left(i\right),Inje\left(j\right)\right)-p^{\prime }\left(Inje^{\prime }\left(i\right),Inje^{\prime }\left(j\right)\right)|\leq \alpha$

2) $\sum_{i=1,j=1}^{k}|{\text{Δ}}_{ij}|\leq \theta$

**Figure 1:**
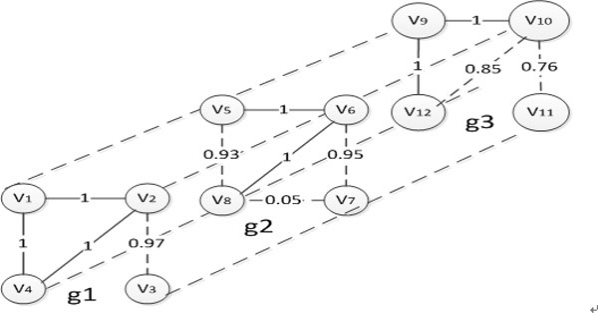
**Example of probability graph isomorphic**.

Then the graph *g *and *g*′ are called probability isomorphic, also denoted as *g *≈ *g*′. **[Example 1] **Shown in Figure 1, giving the probability graphs *g*_1_, *g*_2 _and *g*_3 _, *α *= 0.1, *θ *= 1.0, the optimal node mapping sequence $\left\langle Inje_{1},Inje_{2},Inje_{3}\right\rangle =\left\langle \left\{v_{1},{\text{v}}_{2},v_{3},v_{4}\right\},\left\{v_{5},{\text{v}}_{6},v_{7},v_{8}\right\},\left\{v_{9},{\text{v}}_{10},v_{11},v_{12}\right\}\right\rangle$. Because $|p_{3}\left(v_{10},v_{11}\right)-p_{2}\left(v_{6},v_{7}\right)|>\alpha ,|p_{3}\left(v_{10},v_{11}\right)-p_{1}\left(v_{2},v_{3}\right)|>\alpha$, *g*_3 _and *g*_1 _, *g*_2 _are not probability isomorphic. But *g*_1 _and *g*_2 _fulfil the two conditions in Definition 2, so they are probability isomorphic.

Compared to the certain graph, the probability graph requires two graphs' topology approximated. The probability graph also takes into account the degree of weights matching of the corresponding edges between nodes, so the computational complexity of the probability graph is higher than certain graph isomorphism. On the other hand, the probability adjoint matrix can be used to uniquely identify a probability graph, but a different arrangement of the nodes in the probability graph may correspond to multiple probabilistic adjoint matrixes, resulting in high computational complexity to find the probability graph isomorphism.

**Definition 3 **(Frequent subgraph of probabilistic networks): Probability subgraphs of the node's scale are *k *denoted as $g_{D}^{k}=\left\{g_{1},g_{2},\ldots ,g_{n}\right\}$ (the following texts call it probabilistic graph set), which are obtained from the probability networks, and $sup\left(g_{\alpha },g_{D}^{k}\right)$ means the degree of support for graph *g_α _*in $g_{D}^{k}$.

$$\begin{array}{c} \delta \left(g_{\alpha },g_{i}\right)=\left\{\begin{array}{cc} 1 & g_{a}\;\text{and}\;g_{i}\;\text{is probability isomorphic}\\ 0 & g_{a}\;\text{and}\;g_{i}\;\text{isn't probability isomorphic} \end{array}\right)\\ sup\left(g_{\alpha },g_{D}^{k}\right)=\sum_{g_{i}\in g_{D}^{k}}\delta \left(g_{\alpha },g_{i}\right) \end{array}$$

User-specified minimum support threshold denoted as *min_sup*, suppose frequent pattern set is *F*, if $sup\left(g_{\alpha },g_{D}^{k}\right)\geq min\text{\_}sup$, then *g_α _*∈ *F *is a frequent pattern. Shang etc. [27] proposed an exact graph isomorphism algorithm based on circuit simulation method, which is mainly used in a directed graph, undirected graph and mixed graph (refers as a mixture of directed graph or undirected graph). Therefore, inspired by the method in [27], the probability graph isomorphism algorithm is proposed. The probability graph isomorphism algorithm uses the innovative construction method based on associated circuit and node voltage sequence alignment algorithm. In order to avoid the high computation caused by the differential calculation of every edge (|Δ*_ij_*| ≤ *α*) in the adjoint matrix, we introduce threshold *ε *which is the sum of the corresponding edge difference in associated circuit based on the circuit simulation. Then based on the probability graph isomorphism algorithm, the hierarchical clustering method is adopted to cluster subgraphs to discover frequent probability patterns from the clusters.

So, the method of frequent probability pattern mining in biological uncertain networks based on circuit simulation is defined as the followings (Assume that the node number for mining the frequent probability patterns is *k*):

(1) **Probability subgraph set *g_D _***Since the large numbers of motifs discovered by biological functions are non-tree structure [9], only non-tree subgraphs are necessary to be searched in the biological networks. Therefore, the subgraph search algorithm of non-tree based on the division [28] is used for getting all of candidate probability subgraph set *g_D _*with the size *k *from the biological probability network.

(2) **Probability graph isomorphism evaluation based on the circuit simulation method**. Given the mismatch threshold *ε *of node voltage sequence matrix and the probability mismatch threshold *θ *of the adjoint matrix, two probability graph distance matrix *Dist *(*N, N*′) are obtained using the circuit simulation method. Then the Hungarian Algorithm is used to get the node optimal matching sequence and the mismatch value of node voltage sequence matrix. Based on the mismatch value of node voltages sequence matrix, the node optimal matching sequence and the mismatch value of adjoint matrix, then two probability graphs are evaluated to be probability isomorphic or not.

(3) **Frequent probability subgraph discovered by two-step hierarchical clustering**. According to the method of probability isomorphic introduced in (2), two-step hierarchical clustering is used to find the probability subgraph isomorphism group, and then get the frequent probability subgraphs.

The next sections will introduce the method of probability graph isomorphism evaluation and the algorithm of two-step hierarchical clustering for discovering frequent probability subgraph.

### Probability graph isomorphism judgment based on the circuit simulation method

In this algorithm, the node voltage method [27] of the basic linear circuit analysis method is used.

### The basic linear circuit analysis method- node voltage method

The node voltage method is a circuit simulation method based on the principle of conservation current. The principle of conservation current specifies that the current is unlikely to disappear and it is impossible to suddenly increase, so the amount of current inflow is equal to the amount of current outflow in a closed circuit. Based on this principle, the circuit voltage can be calculated.

In the circuit network, a node is arbitrarily selected as the reference node. The electric potential difference between each of the remaining nodes and the reference node is known as the voltage of the node. Obviously, the number of node voltages is less than a number of nodes. For a *k*-node network, there are (*k*-1) node voltages. For a *k*-node network, node *k *is taken as the reference node, and then the node voltage equation can be expressed as:

(1) $$\left(\begin{array}{c} \;\;G_{11}U_{k1}+G_{12}U_{k2}+\ldots +G_{1\left(k-1\right)}U_{k\left(k-1\right)}=I_{s1}\\ \;\;G_{21}U_{k1}+G_{22}U_{k2}+\ldots +G_{2\left(k-1\right)}U_{k\left(k-1\right)}=I_{s2}\\ \;\;\ldots \ldots \ldots \\ G_{\left(k-1\right)1}U_{k1}+G_{\left(k-1\right)2}U_{k2}+\ldots +G_{\left(k-1\right)\left(k-1\right)}U_{k\left(k-1\right)}=I_{s\left(k-1\right)} \end{array}\right\}$$

Using matrix is represented as:

(2) $$\left[\begin{array}{cccc} G_{11} & G_{12} & \cdots \; & G_{1\left(k-1\right)}\\ G_{21} & G_{22} & \cdots \; & G_{2\left(k-1\right)}\\ \cdots \; & \cdots \; & \cdots \; & \cdots \\ G_{\left(k-1\right)1} & G_{\left(k-1\right)2} & \cdots \; & G_{\left(k-1\right)\left(k-1\right)} \end{array}\right]\left[\begin{array}{c} U_{k1}\\ U_{k2}\\ \cdots \\ U_{k\left(k-1\right)} \end{array}\right]=\left[\begin{array}{c} I_{s1}\\ I_{s2}\\ \cdots \\ I_{s\left(k-1\right)} \end{array}\right]$$

Where *G_ii _*(*i *= 1, 2,..., *k *− 1) is called the self-admittance of node *i*, whose value is the sum of the admittance of all branches connected to the node *i*.

*G_ij _*(*i *= 1, 2,..., *k *− 1; *j *= 1, 2,..., *k *− 1) is called mutual admittance of node *i *and node *j*, which is the negative of the sum of all branches' admittance between the node *i *and node *j *.

*U_ij _*is the voltage of the node *j *when node *i *is selected as reference node.

*I_si _*(*i *= 1, 2,..., *k *− 1) is the algebraic sum of the current flow into node *i *(inflow is positive, outflow is negative).

**Figure 2:**
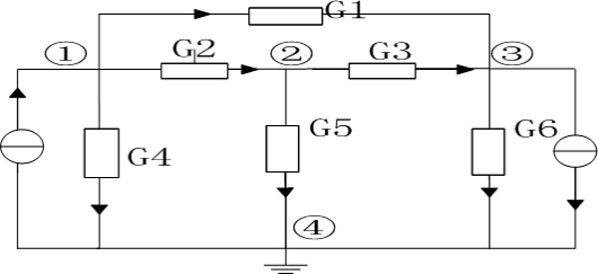
**Example of node voltage method**.

**[Example 2] **The circuit *N *is shown in Figure 2. When the node ④ is denoted as a reference point, *U*_41_, *U*_42_, *U*_43 _are the voltage difference between the three nodes ①, ②, ③ and node ④ respectively, also are the voltage values of node ①, ②, ③. Using the above method of node voltage, we can get the following equations:

$$\left[\begin{array}{ccc} G_{1}+G_{2}+G_{4} & -G_{2} & -G_{1}\\ -G_{2} & G_{2}+G_{3}+G_{5} & -G_{3}\\ -G_{1} & -G_{3} & G_{1}+G_{3}+G_{6} \end{array}\right]\left[\begin{array}{c} U_{41}\\ U_{42}\\ U_{43} \end{array}\right]=\left[\begin{array}{c} I_{s1}\\ 0\\ -I_{s2} \end{array}\right]$$

By solving these equations, we can get the node voltage sequences[*U*_41_, *U*_42_, *U*_43_], when node ④ is the reference point.

In Example 2, we can see that, set a node (e.g. ④) as a reference point, the each node voltage values of the sequence (e.g. *U*_41_) is calculated based on the information of topology and edge weight (e.g. when solving *U*_41_, take into account the conductance values of *G*_1_, *G*_2 _and *G*_3 _,which are adjacent to node ① etc.). From this perspective, to some extent, the node voltage sequences characterize the topology information and edge weight information of the probability adjacency matrix.

### Node voltage method for probability graph

**Definition 4 **(associated circuit of probability graph): For graph *g*, if the reciprocal of probability (1/ *p, p *∈ (0,1]) of each edge is used to represent the resistance value (i.e. when the edge probability value is close to 0, the circuit is disconnected, indicating that the circuit has little effect on the whole graph for node voltages), then we get the circuit *N *called the associated circuit of graph *g*.

**Figure 3:**
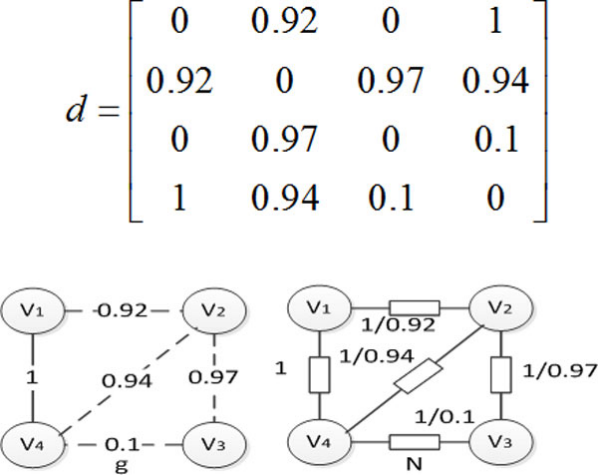
**Probability graph *g*, adjoint matrix *d *and associated circuit *N***.

**[Example 3] **Figure 3 shows the probability graph *g*, its corresponding adjoint matrix *d *and its associated circuit *N*.

By the definition, *G_ij _*of equation (1) and (2) is expressed as conductance, the relationship equation between conductance and resistance is *G *= *1/R *for purely resistive circuit. Here set *R = 1/p*, then *G = p*, where *R *represents resistance and *p *represents the probability value of edges in the probability graph. When *p *→ 1, which means that the circuit is connected with a very small resistance, the circuit is close to zero resistance; when *p *→ 0, which means that the circuit is connected with an infinite resistance, the circuit is close to disconnected.

**Definition 5 **(similar circuit): Let us define the corresponding topology graph of the circuit *N *as *g *and the corresponding topology graph of the circuit *N*′ as *g*′. If *g *and *g*′ are the approximate isomorphic graph, then the corresponding branches of *N *and *N*′ contain similar resistance value elements, and *N *and *N*′ is called similar circuit, denoted as *N *≈ *N*′.

**Theorem 1: **Two of associated circuits based on their probability isomorphism subgraphs are similar circuits, i.e. if *g *≈ *g*′, then *N *≈ *N*′.

**Proof: **Since the probability isomorphism subgraphs have similar topology *g *and *g*′, and each edges of *g *and *g*′ are replaced with similar resistance, associated circuit has similar elements corresponding to their branches. Therefore, *N *and *N*′ is similar circuit.

Two of associated circuits *N *and *N*′ based on their isomorphism probability subgraphs *g *and *g*′ are similar circuits. On the contrary, if two of the associated circuit *N *and *N*' are similar circuits, it could not be directly concluded that two probability subgraphs *g *and *g*′ of these associated circuit are probability isomorphism. Thus, the necessary condition of probability isomorphism for two probability subgraphs is that the associated circuits of probability subgraphs are similar circuits.

**Definition 6 **(complete excitation [27]): Associated circuit *N *with *k *nodes, let node *I *be a reference node, apply the same current source *I_s _*(the value of the current source *I_s _*is taken as 1A without loss of generality) between node *i *and the remaining (*k*-1) nodes, respectively, with the directions of the currents being from node *i *to the other nodes. This kind of excitation is called a complete excitation of node *i*.

**Figure 4:**
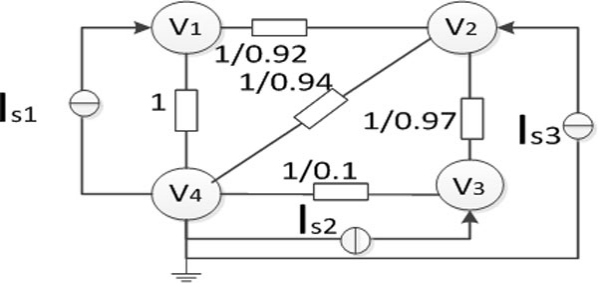
Complete excitation of node *V4*.

According to the method of complete excitation [27], the associated circuits of probability subgraph with complete excitation of the nodes are obtained. For example, Figure 4 shows the complete excitation of the node *V*_4 _as a reference point from Figure 3.

**Definition 7 **(Node voltage sequence [27] and node voltage sequence set [27]):

In an associated circuit *N*, when the node *i *serves as a reference point, the set of node voltages in an ascending order is obtained based on the complete excitation of node *i*. This set is called the node voltage sequence of node *i*, denoted as *S_i_, i *= 1, 2, ..., *k *(*k *is the number of nodes in the associated circuit). Furthermore, the node voltage sequences of all the nodes constitute the node voltage sequence set of the circuit *N*, denoted as *S *= {*S*_1_,..., *S_i_*}*^T^, i *= 1, 2, ..., *k*.

**[Example 4] **For probability graph *g *in Figure 3, according to the node voltage method set the node *V*_4 _as a reference point, where the resistance *R = 1/p*, the conductance *G = p*, we get the *G*_4 _as following:

$$G_{4}=\left[\begin{array}{ccc} 1+0.92 & -0.92 & 0\\ -0.92 & 0.92+0.97+0.94 & -0.97\\ 0 & -0.97 & 0.1+0.97 \end{array}\right]$$

According to the formula 2, the node voltage equations: *GU=I*, the node voltage sequence *S*_4 _is obtained as follows:

$$S_{4}=\left[1.277978\;1.580127\;2.367031\right]$$

The rest can be done in the same manner, so the node voltage sequence set of circuit *N *are obtained as follows:

$$\begin{array}{c} \text{S}={\left\{S_{1},\;S_{2},\;S_{3},\;S_{4}\right\}}^{T}\\ S=\left[\begin{array}{ccc} 1.413642 & 1.724302 & 2.629848\\ 1.034504 & 1.069194 & 1.077705\\ 2.726954 & 3.548548 & 3.675701\\ 1.277978 & 1.580127 & 2.367031 \end{array}\right] \end{array}$$

**Theorem 2: **For two of associated circuits *N *and *N*′ based on their probability isomorphism subgraphs graph *g *and *g*′, if the corresponding nodes have the same complete excitation, the corresponding nodes' voltage are similar.

**Proof: **Assume that *d *and *d*′ are the adjoint matrix of the probability isomorphic graph *g *and *g*′, so *d *≈ *d*′. Construct the associated circuits *N *and *N*′ respectively, according to the definition of associated circuits, *N *and *N*′ is similar circuit, denoted as *N *≈ *N*′. Let *Y_b _*and ${Y_{b}}^{\prime }$ are branch admittance matrixes respectively, then $Y_{b}\approx {Y_{b}}^{\prime }$.

Select the corresponding node *i *and *i′ *of probability isomorphic as the reference point respectively, and the complete excitation *I_s _*and ${I_{s}}^{\prime }$, then the node voltage equations [27] of *N *and *N*′ are:

$$\left\{\begin{array}{c} dY_{b}d^{T}U_{k}=dI_{s}\\ d^{\prime }\;{Y_{b}}^{\prime }{d^{\prime }}^{T}\;{U_{k}}^{\prime }=d^{\prime }\;{I^{\prime }}_{s} \end{array}\right)$$

Where *U_k _*and $U_{k}^{\prime }$ are the node voltage sequence for node *i *and *i*′ which are the reference point respectively. Because the complete excitation is same i.e. $I_{s}={I_{s}}^{\prime }$, $U_{k}\approx {U_{k}}^{\prime }$. The theorem is proved.

**Theorem 3: **If *g *and *g*′ are probability isomorphic, the node voltage sequence set of each of associated circuits *N *and *N*′ in the same complete excitation are similar, denoted as *S *≈ *S*′. So that the mismatch value of the two group of node voltage sequence set is less than the threshold value *ε *,denoted as |*S *− *S*′| <*ε*.

**Proof: **If *g *and *g*′ are probability isomorphic, the node voltage sequences of the node *i *and node *i*′ in each of associated circuits *N *and *N*′ in the same complete excitation are correspondingly similar. And if *g *and *g *' are probability isomorphic, according to Definition 2, the absolute value of weight difference of the corresponding edges between nodes is less than or equal to the threshold value, i.e. |Δ*_ij_*| ≤ *α*. Based on Theorem 2, $U_{ij}\approx {U_{ij}}^{\prime }$ can be obtained, i.e. $\left|U_{ij}\approx {U_{ij}}^{\prime }\right|$ is less than a threshold value denoted as *ε_ij _*, therefore $\sum_{i,j=1}^{k}|U_{ij}-{U^{\prime }}_{ij}|<\sum_{i,j=1}^{k}\varepsilon_{ij}$, let $\sum_{i,j=1}^{k}\varepsilon_{ij}=\varepsilon$, then the mismatch value of two node voltage sequence set is less than the threshold i.e. |*S *− *S′*| <*ε *. The theorem is proved.

Based on the analysis of the circuit simulation method for determining the graph probability isomorphic, we make the conclusion that the mismatch value of two graphs' nodal voltage matrix being less than the probability threshold is the necessary condition of probability isomorphic of two graphs. So, next we will discuss how to get the mismatch value of two graphs' nodal voltage matrix.

### Hungarian algorithm for optimal node matching in isomorphic graph decision

Suppose *S *and *S*′ are the node voltage matrix of probability graph *g *and *g*′, *k *is graph node size of *g *and *g*′, so there is *k *kind of possible node mapping relations of *S_i _*(*i *= 1,..., *k*) and *S_j _*(*j *= 1,..., *k*), *S_i _*and *S_j _*is the node voltage sequence in corresponding *g *and *g*′. The *k *nodes of the node voltages sequence in *S *may have *k*! possible mapping relations corresponding to *S*′. Therefore, in order to determine probability isomorphic of two probability graphs, we need to evaluate whether the mismatch value of two graphs' nodal voltage matrix is less than the threshold. In other words, we need to find the best node mapping relation of two probability graphs so that the mismatch value of two graphs' nodal voltage matrix is minimum and less than the threshold. The mismatch value of two graphs' nodal voltage matrix can be calculated by the distance of node voltages sequence between any two nodes in the distance matrix. *Dist *is defined as formula 3.

(3) $$Dist=\left|A_{KK}-B_{KK}\right|=\sqrt{diag\;\left[A_{KK}*A_{KK}\prime \right]*{\left[1\right]}_{K^{*}K}+diag\left[B_{KK}*B_{KK}\prime \right]*{\left[1\right]}_{K^{*}K}-2*A_{KK}*B_{KK}\prime }$$

Thus, the problem of seeking the optimal node mapping between graphs is converted to the problem of finding the minimum of sum of elements of rows and columns in distance matrix *Dist*. This problem is a classic bi-graph matching problem, and can be solved by Hungarian algorithm [29] as an assignment problem.

Hungarian algorithm is mainly based on the following facts: if each element of a row (or a column) in the coefficient matrix *C *= (*c_ij_*) is added or subtracted by the same number to get a new matrix *B *= (*b_ij_*), the assignment problem with the coefficient matrix *C *and *B *has the same optimal assignment.

**Definition 8 **(node voltage sequence matrix mismatch value *VMval*): Given probability graph *g *and *g*′, and the node voltage sequence matrix *S *and *S*′ which are associated circuits *N *and *N*′ respectively, according to *S *and *S*′, get distance matrix *Dist *of node voltage sequence and use the Hungarian algorithm to obtain assignment matrix *M*. In the *Dist *matrix, the sum of the elements at the corresponding location with the value of "1" in the assignment matrix *M *is equal to the minimum mismatch of two node voltage sequence matrix, and this value is noted as *VMval*.

**Definition 9 **(adjoin matrix mismatch value *PMval *of probability graph): Given *g *and *g*′, according to *S *and *S*′ obtain distance matrix *Dist *of node voltage sequence and use the Hungarian algorithm to obtain assignment matrix *M*, then get the node mapping sequence <*Inje, Inje*' > of two probability graph, and the adjoin matrix *d *and *d*′ are obtained by <*Inje, Inje*' >, *PMval *is equal to the absolute value of the sum of corresponding edge weights difference in the adjoin matrix *d *and *d′ *adjusted, i.e. $PMval=\sum_{i,\;j=1}^{k}\left|p\left(Inje\left(i\right),Inje\left(j\right)\right)-p^{\prime }\left(Inje^{\prime }\left(i\right),Inje^{\prime }\left(j\right)\right)\right|$

Based on the node mapping relationship <*Inje, Inje*' > obtained from Hungarian algorithm, then perform elementary transformation for matrix *d *and *d*′ respectively, called matrix adjust, where matrix *d *performed the elementary transformation by *Inje *, the matrix *d*′ performed the elementary transformation by *Inje*′.

### Algorithm of probability subgraph isomorphism

The necessary conditions of two probability subgraphs isomorphism is that the mismatch value of their associated circuit node voltage sequence set is less than the threshold *ε *. So, if the mismatch value *VMval *of node voltage set exceeds the threshold value *ε , g *and *g*′ is not isomorphic. If the mismatch value *VMval *less than *ε *,then according to the Definition 2 of probability graph isomorphism, the mismatch value of the corresponding edge of two probability subgraphs should be less than the threshold value *α *and the sum of mismatch value of corresponding edges of probability subgraphs should be less than the threshold value *θ *. Because of high computational cost to evaluate each mismatch value of edges adjoint matrix, we add the mean and variance of node voltage sequences as the additional column in the distance matrix of node voltages sequence set when we calculate the mismatch value *VMval*. Thus, the mismatch value *VMval *is somewhat similar to the mismatch value of the corresponding edges.

Assume *k *is the number of nodes of probability graph. The probability adjoint matrix can be used to uniquely identify a probability graph, but a different arrangement of the nodes in probability graph may correspond to *k *! probabilistic adjoint matrix. Since the Hungarian algorithm can get one of optimal assignment matrix *M*, if *PMval> θ *based on the *M*, it illustrates that this mapping relationship may be not correct and two graphs are still probability isomorphic. So, *k *! -1kind of elementary matrix transformation for adjusting adjoint matrix *d *and *d*′ may be necessary to discover whether two probability graphs are isomorphic or not.

Thus, the main steps of the algorithm to discover the probability subgraph isomorphism are as follows:

• Firstly, according to the probability graph *g *and *g*′, get the associated circuit *N *and *N*′, then calculate the node voltages sequence matrix *S *and *S*′; Next, using the Hungarian algorithm to get the assignment matrix *M *and the mismatch values *VMval*. If the mismatch value *VMval *of node voltage set exceeds the threshold value *ε *, that is to say, *g *and *g *' are inevitably not isomorphic, otherwise, go to step 2

• According to "1" in the column coordinate of assignment matrix *M*, generate node mapping relationship, then according to the node mapping relationship <*Inje, Inje*' > of node voltage sequences set in *N *and *N*′, adjust adjoint matrix *d *and *d*′. If the mismatch value of adjoint matrix *PMval *<*θ *, then *g *and *g*′ are probability isomorphic, otherwise, they are possibly isomorphic, go to step 3

• In this case, *k*!-1 kind of elementary matrix transformation of adjoint matrix *d *and *d*′ may be needed to discover whether two probability graph are isomorphic or not. When there is a new mapping satisfying *PMval *<*θ *, the two graphs *g *and *g *' are probability isomorphic; on the contrary, they are not isomorphic.

The pseudocode of algorithm of probability graph isomorphism evaluation based on circuit simulation is shown in Table 1.

**Table 1:** Algorithm of probability graph isomorphism judgment based on circuit simulation.

**Algorithm**: Isomorphism judgment Algorithm of probability graph **IsomorphismCal **(*g_α _, g_i _, ε , θ*) **Input**: two probability graph *g_α _*and *g_i_*, the mismatch threshold value *ε *of the node voltage sequences set, the mismatch threshold value *θ *of probability adjoint matrix **Output**: the bool value *Iso *of two graph isomorphism, the node mapping sequence *Inje_i_*, the mismatch value *VMval_i _*of node voltage sequences matrix
1. **//Generate the node voltage sequence matrix ***S_α _, S_i _***according to ***g_α _, g_i_* **For **each graph in {*g_α _, g_i_*} generate associated circuit *N_α _*and *N_i_* **End** **For **each graph in {*g_α _, g_i_*} **For ***j *= 1 to *k *Calculate the node voltage sequence *S ^j ^*while set *node_j _*as reference node; $S=S^{J}\cup S,S\in \left\{S_{\alpha },S_{i}\right\}$ **End** **End** 2. **//calculate distance matrix according to ***S_α _, S_i_* **Calculate ***Dist*(*N_α_, N_i_*) according to formula(3); 3. **//Get the node mapping relationship ***Inje_i _***and the mismatch value of node voltage ***VMval_i _***by Hungarian algorithm** *VMval_i _, Inje_i _*> ← *Hungarian*(*Dist*); 4. // **Compare ***VMval_i _***and ***ε* **If ***VMval_i _*<= *ε* *g_α _*and *g_i _*is not probability isomorphic; ** Continue**; **Else** *g_α _*and *g_i _*maybe probability isomorphic; // *g_α _*and *g_i _*maybe probability isomorphic, and need further deal **5. //Calculate the mismatch value of adjoint matrix ***PMval_i _***by adjust the adjoint matrix of ***g_α _***and ***g_i _***according to nodes mapping sequence** **If ***PMval_i _*<= *θ* *g_α _*and *g_i _*is probability isomorphic; *Iso *=True; **Return **<*Iso, Inje_i _*,*VMval_i _*> ; **Else** **Return **PermuteInjectedSequenceAndTestIso (*g_α_, g_i_, Inje_i_, VMval_i_, θ*); // Enumeration the node mapping relationship for isomorphic judgment **End**

The algorithm of probability graph evaluation based on possible world model has *O*(2^|*E*|^) of the best time complexity and *O*(2^|*E*| ^* *k*!) of the worst time complexity (|*E*| is the number of edges in probability graphs). The algorithm of probability graph isomorphism evaluation based on circuit simulation method excludes most of subgraphs which are not probability isomorphism and reduces the search space of the probability isomorphism subgraphs using the mismatch value *VMval *of node voltage set. In the narrowed set of subgraphs, the mismatch values of its subgraphs are calculated by the enumeration method. The best time complexity of the algorithm is *O*(1), i.e. the minimum mismatch sequence of node voltage sequence matrix is the mapping sequence of probability isomorphism, the worst time complexity of which is *O(k!)*, when we need to enumerate all possible nodes mapping relationship.

### Frequent probability pattern identifying algorithm

Based on the method of probability subgraph isomorphic, the frequent probability pattern can be identified from the probability subgraph set using graph alignment. Usually, the Star-alignment is adopted for frequent pattern identified in certain graph. It needs to traverse the entire graph set as the centre graph for comparison leading to high complexity. In this paper, the algorithm of two-step hierarchical clustering is proposed for frequent probability pattern identification in order to effectively reduce the computational complexity.

In the process of the traditional hierarchical clustering, every cluster is selected based on the two subgraphs with minimum distance in all classes, to some extent, which ensure two probability graphs are the most similar in each cluster. However, it's time complexity is *O(n*^3^*)*, which is not suitable for large-scale data processing. Meantime, *n × n *similarity matrix should be stored, so that it occupies a large amount of the memory space. As isomorphic evaluation between two probabilities subgraphs are based on their mismatch value of *VMval *and *PMval *. Therefore, the algorithm of two- step hierarchical clustering consists of two major steps. Firstly, similar to the method of merging clustering, two probability graphs are clustered as long as they fulfill the threshold of probability isomorphic and they need not be the two subgraphs with minimum distance. This process continues until the distance between any of two clusters is less than the mismatch threhold. Then, the idea of classical hierarchical clustering is adopted by the algorithm to group the two clusters with smallest distance in all clusters. The algorithm terminates until clustering distance of any two subgraphs surpass their mismatch threshold. The pseudocode of the algorithm of frequent probability subgraph discovered by two-step hierarchical clustering is shown in Table 2.

**Table 2:** Algorithm of frequent probability pattern by two-step hierarchical clustering.

**Algorithm: **Two-step Hierarchical Clustering For FPP (*G, ε, θ ,freq*) **Input**: All probability subgraphs with *k *scale **Output**: frequent probability subgraph *g_α_*
1. **Initilize **the *n *graphs {*g*_1_,...,*g*_n_}**as **the n leaves of cluster tree ; 2. **While **Change_*label*!=0 3. Change_*label *= 0; // Change_*label *indicates whether the process of merging clustering operation 4. *L*_c _= size(ResidentGraph); //Calculation subgraph number, *L*_c _represents the total number of clusters 5. **For ***i*= 1 **to ***L*_c _/2 6. <*Iso,inje,VMval*>=IsomorphismCal(*g*_i_, *g*_i+ *L*c /2 _, *ε, θ*); // Determine *g*_i_, *g*_i+ *L*c /2_probability isomorphic 7. **If ***Iso*= =**TRUE** 8. *g*_i _= union(*g*_i_, *g*_i+ *L*c /2_); 9. Change_*label *++; 10. ResidentGraph = {ResidentGraph *i*}; //if isomorphic, retaining only the subgraph *label i *to ResidentGraph 11. **Else** ResidentGraph = {ResidentGraph *i i+ L_c _/2*};//if not isomorphic, retaining only the subgraph *label i, i+ L_c _/2 *to ResidentGraph 12. **End if** 13. **End for** 14. **End while** 15. SimpleHierarchicalClusteringForFrequentSubgraphWithPro(ResidentGraph, *ε, θ ,freq*); //using a simple hierarchical clustering for the remaining probability subgraphs 16. Calculate the probability isomorphic frequency *p *of the residual clusters *g*_r _;

The algorithm of frequent probability subgraph discovered by two-step hierarchical clustering takes *L_c_*/2 as each step for the comparison (*L_c _*is the number of cluster), so that the new categories are the most likely to become the two candidate classes for pairwise comparison at next step of clustering. This approach avoids the poor clustering results led by little change of two candidate classes when searching for clustering. The algorithm of frequent probability subgraph discovered by two-step hierarchical clustering takes *L_c_*/*2 *as each step and each step compares the mismatch value of node voltage sequences matrix *VMval *with the mismatch value of probability isomorphic of adjoint matrix *PMval *obtained from *g_i _*and $g_{i+L_{c}/2}$. When they satisfies the conditions of *VMval *<*ε *∩ *PMval *<*θ, g_i _*and $g_{i+L_{c}/2}$ are combined directly. This process is reduced into classical hierarchical clustering until the mismatch value between any of two probability subgraph at interval step *L_c_*/2 are greater than <*ε, θ *>.

As can be seen from the above analysis, the time complexity of frequent probability subgraph recognition algorithm based on two-step hierarchical clustering is *O *(*n *log *n*) in the best case, and the time complexity in the worst case is *O(n*^3^*) *since the algorithm is reduced to a classical hierarchical clustering. In the space complexity, as a result of using a simple hierarchical clustering and the subgraph index set "ResidentGraph" to reserve and update the clusters of subgraphs, the distance matrix *n *× *n *spatial complexity consumption is avoided. However, in this approach, the mismatches of distance between any two subgraphs are required to calculate at each clustering round at the expense of time consumption.

In addition, because the distance between any of two subgraphs in each hierarchical clustering may not the smallest, it reduces the convergence time of clustering and may cause clustering bias. This bias is limited by probability isomorphic threshold, i.e. the greater the probability isomorphic threshold is, faster two-step hierarchical clustering cluster runs with the larger deviation of clusters; Conversely, the smaller the probability isomorphic threshold is, slower hierarchical clustering method runs with less small deviation clustering. Fortunately, the isomorphism probability threshold is defined by the user, so the error caused by the hierarchical clustering is limited within the acceptable range to users. Therefore, compared with the classical hierarchical clustering, the algorithm of frequent probability subgraph discovered by two-step hierarchical clustering has good clustering results with the significantly lower time complexity.

## Experimental setup and result analysis

To verify and evaluate the performance of the proposed algorithm in this work, three real-world biological networks are used, including the transcriptional regulatory network of *E.coli *[30], transcriptional regulatory networks of *S.cerevisiae *: based on the ChIP-chip data[31,32] called *S.cere^1 ^*dataset and *S.cere^2 ^*dataset respectively. *E.coli *dataset having an exact graph with direction contains 423 nodes and 519 edges, *S.cere^1 ^*data set with a probability of network contains 2428 nodes and 4348 edges, and *cere^2 ^*data set, a probability network, contains 3799 nodes and 13155 edges. The algorithm is applied to *E.coli *data set to validate the correctness and performance of probability isomorphic algorithms, using exact graphs. And the algorithm is applied to *S.cere^1 ^*and *S.cere^2 ^*datasets to verify frequent subgraph mining in probabilistic networks. The verification process is based on whether the generated results in this study contain motifs which were reported in other research works [10,11].

### Demonstration and comparison of probability isomorphism algorithm

**Figure 5:**
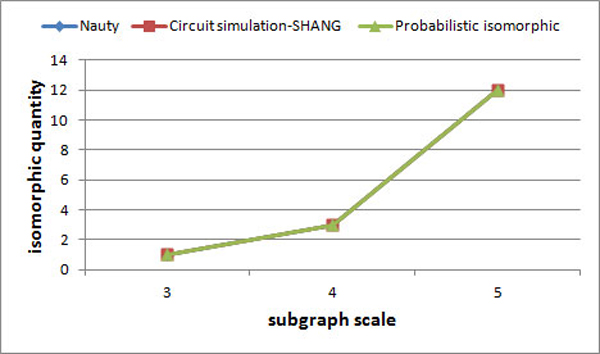
**Results of three graph isomorphism algorithms with *E.coli *data**.

The algorithm of exact graph isomorphism is a special case of probabilistic isomorphic algorithm when the probability of edge is 1 or 0, so the algorithm for probability isomorphism is also applicable for exact graph isomorphism algorithms with the mismatch values of *VMval *and *PMval *equal to zero. Firstly,the partitioning based non-treelike subgraph mining algorithm were used to efficiently search non-treelike subgraphs from *E.coli *data set. Then, the classic graph isomorphism algorithm Nauty[33], the exact graph isomorphism algorithm based on circuit simulation proposed by SHANG[27] and probability isomorphic algorithms proposed in this paper were performed on the data set of non-treelike subgraphs. Nauty is implemented by C++, while the other two are implemented by matlab, because of more matrix calculation. The scale of Non-tree subgraph, the Number of subgraphs and the Number of subgraph isomorphism classes are shown in Table 3 and the results of three graph isomorphism algorithms with *E.coli *data is shown in Figure 5.

**Table 3:** The relationship of Non-tree subgraph scale, Number of subgraphs and Number of subgraph isomorphism Classes from *E. coli *data.

*subgraph scale*	*3*	*4*	*5*
number of subgraphs	42	1822	57632
number of subgraph isomorphism classes	1	3	12

**Figure 6:**
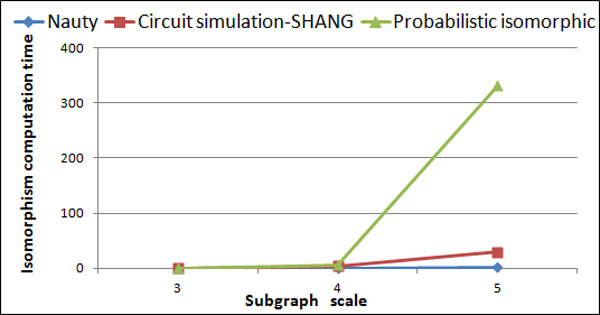
**The Performance of three graph isomorphism algorithm on *E.coli *data**.

From Figure 5, it can be seen that the probabilistic isomorphic algorithm is correct when it is applied for exact graph isomorphism. As shown in Figure 6, the performance of the existing classical algorithm of exact graph isomorphism is superior to probabilistic isomorphic algorithms proposed in this paper. Meanwhile, we found that the performance of algorithm proposed by SHANG circuit-based simulation of certain graph isomorphism is significantly better than the probability isomorphic algorithm proposed in our paper when performing the task of identifying five scale subgraphs. The main reason is that the algorithm of exact graph isomorphism does not need to calculate the distance matrix and compute the node mapping sequence using the Hungarian algorithm. It just needs to compare whether the node voltage sequences are equal correspondently (rather than approximate), so the time complexity of certain graph isomorphism is smaller than probability isomorphic algorithms. However, the proposed method is designed to calculate a group of graphs for probability isomorphic directly instead of finding the solution to certain graph isomorphism. By experimenting isomorphism on certain graph set, we can prove that the probability isomorphism theory based on circuit simulation method is feasible.

### Verification of frequent probability subgraph by two-step hierarchical clustering

In this experiment, the algorithm of frequent probability subgraph by two-step hierarchical clustering was tested on *S.cere^1 ^*and *S.cere^2^*. In order to be compared with the result in the paper [10,11], frequent probability subgraph with 3-4 scale nodes and 5 scale nodes were identified in *S.cere^1 ^*and *S.cere^2 ^*separately.

**Figure 7:**
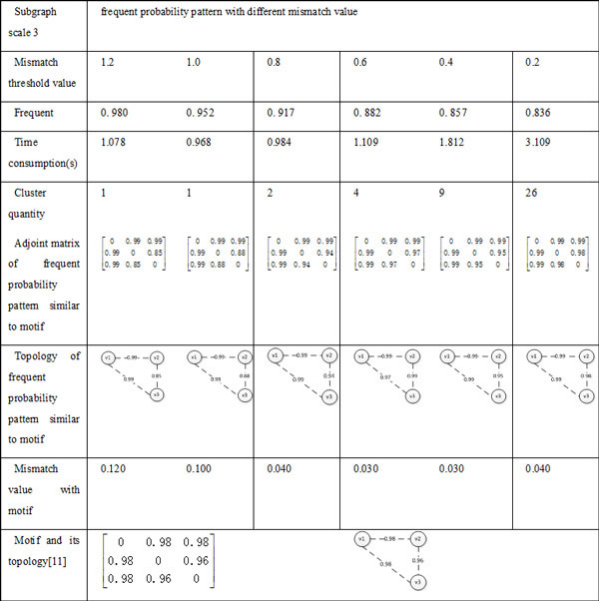
**Comparison of frequent probability pattern and motif with 3-scale subgraph**.

**Figure 8:**
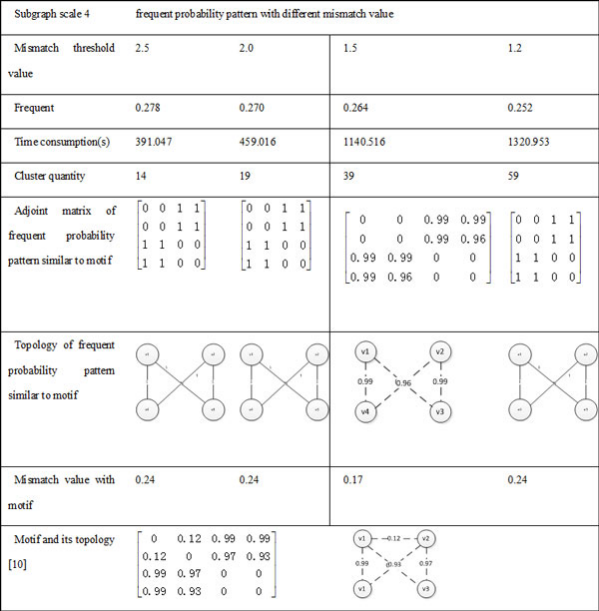
**Comparison of frequent probability pattern and motif with 4-scale subgraph**.

**Figure 9:**
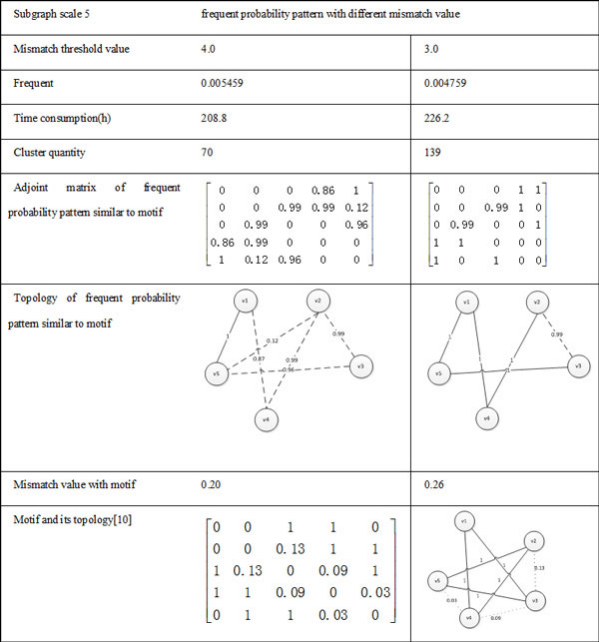
**Comparison of frequent probability pattern and motif with 5-scale subgraph**.

In the algorithm of frequent probability subgraph using two-step hierarchical clustering, the mismatch value *ε *and *θ *need to be set. Based on experiment results, we discover that when *ε *remain constant, *θ *reduces and the number of clusters increases. When *θ *remain constant, *ε *reduces and the number of clusters increases too. That is to say, the number of clusters with *ε *and *θ *keeps negative relevance. We also found that for the subgraph with *k *scale, when upper limit value of *ε *and *θ *is *ln(k(k - 1)) *, the number of recognized clusters and results of cluster are more satisfactory (the frequent probability subgraph recognized contains probability motif with smaller number of clusters). Therefore, in the following experiments, we will give the experiment results under the condition of the upper limit of threshold value being *ln(k(k - 1)) *and *ε *= *θ *. When the threshold value changed, the number of clusters and the degree of frequency also changed. To verify that discovered frequent probability patterns include reported motifs, we did not set frequence degree in the process of clustering. The parameters of algorithm are set as follows:

(1) *N *= 3: *ε *-*table *= *θ *− *table *= {1.2,1.0, 0.8, 0.6, 0.4, 0.2} ;

(2) *N *= 4 : *ε *-*table *= *θ *− *table *= {2.5, 2.0,1.5,1.2} ;

The comparison of frequent probability pattern and motif with 3-scale subgraph and 4-scale subgraph are shown in Figure 7 and 8 respectively. In data sets of *S.cere^2^*, as the experiments of the subgraph with 5-scale nodes has a huge amount of data. For example, the number of 5-scale subgraph is 16,372,915, so we have six single machines running at the same time, results shown in Figure 9.

As can be seen from the Figure 7, Figure 8 and 9, discovered frequent probability subgraph contain motifs which were published in others research works [10,11], and the mismatch value with motif are small. Experimental results also indicate that as the clustering threshold mismatch decreases, the number of clustered subgraphs increases, and the frequency of frequent probability pattern similar to motif was gradually reduced, the time of recognizing frequent probability subgraph was increasing. In addition, we found that the discovered frequent probability pattern similar to motif didn't make positive or negative changes with the mismatch value of clustering. The probability subgraph with the highest frequent degree does not necessarily correspond to the smallest mismatch value of motif. Which also proved that motif is not necessarily the most frequent subgraph in the original probabilistic networks, but the subgraph in a original probability with much greater frequency than that in a random network. Therefore, in the probability motif recognizing problem, we also need to have further calculations to get frequency of subgraph in random networks, and then to evaluate whether it is a probability motif.

**Figure 10:**
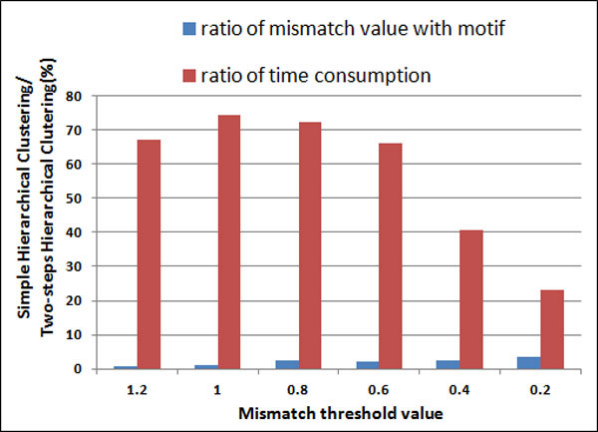
**The ratio of Simple Hierarchical Clustering and Two-step Hierarchical Clutering on the mismatch value with motif and the time consumption**.

Figure 10 compares the time consumption between simple hierarchical clustering method and two-step hierarchical clustering method based on different mismatch threshold values. It indicates that although the mismatch value of frequent patterns and motifs discovered by two-step hierarchical clustering is little larger than the simple hierarchical clustering method, two-step hierarchical clustering has good clustering results with the significantly lower time complexity.

## Conclusions

With the rapid development of biotechnology, especially in high-throughput technologies, a large number of biological network graph data has been produced. It has become a hot topic of current research to identify network substructures associated with a specific function module from this kind of biological networks with intertwined topology and complex functions. Since biological network data carries inevitably experimental error and noisy data, the mining of probability motif in biological probabilistic network will become more practically significant. A key step of identifying probability motif is the mining of frequent probability pattern. This paper presents a probability frequent subgraph mining algorithm in biological probabilistic networks based on the circuit simulation method. A probability frequent subgraph mining algorithm includes the circuit simulation method to evaluate probability isomorphism and two-step hierarchical clustering to recognize frequent probability pattern. Instead of using the possible world model with the exponential time complex, probability isomorphism judgment method combines circuit topology structure and related physical properties of voltage to directly evaluate the probability isomorphism between probability subgraphs. The algorithm of probability graph evaluation based on circuit simulation method excludes most of subgraphs which are not probability isomorphism and reduces the search space of the probability isomorphism subgraphs by the mismatch value of node voltage set. In the narrowed set of subgraphs, the mismatch values of its subgraphs are calculated by the enumeration method.

Furthermore, a frequent probability pattern recognition algorithm based on two-step hierarchical clustering was also proposed for better recognition performance. Experimental results show that the proposed method can produce the satisfactory results, which are consistent with the relevant algorithms. In the future research, the effective approach to solve the problem of symmetrical graph will be further studied because there are several possible mapping sequences for the symmetrical graph, and it will take long time if only enumeration method is used to obtain mapping sequence matches for node adjoint matrix during the calculation.

## Competing interests

The authors declare that they have no competing interests.

## Authors' contributions

JH supervised the work, and JH and WZ contributed to the problem formulation and paper writing. JH and KQ conducted research on the algorithms, and KQ, CW developed and implemented the algorithms. The manuscript was drafted by JH and CW. All authors read and approved the final manuscript.

## Declarations

Publication for this article has been funded by the Natural Science Foundation of Jiangsu Province under Grant No. BK2012742.

This article has been published as part of *BMC Systems Biology *Volume 8 Supplement 3, 2014: IEEE International Conference on Bioinformatics and Biomedicine (BIBM 2013): Systems Biology Approaches to Biomedicine. The full contents of the supplement are available online at http://www.biomedcentral.com/bmcsystbiol/supplements/8/S3.
